# Formation of functional gap junctions in amniotic fluid-derived stem cells induced by transmembrane co-culture with neonatal rat cardiomyocytes

**DOI:** 10.1111/jcmm.12056

**Published:** 2013-05-02

**Authors:** Jennifer Petsche Connell, Emily Augustini, Kenneth J Moise, Anthony Johnson, Jeffrey G Jacot

**Affiliations:** a Department of Bioengineering, Rice UniversityHouston, TX, USA; b Department of Obstetrics and Gynecology, Baylor College of MedicineHouston, TX, USA; c Texas Children's Fetal Center, Texas Children's HospitalHouston, TX, USA; d Congenital Heart Surgery Services, Texas Children's HospitalHouston, TX, USA

**Keywords:** amniotic fluid, cardiac, differentiation, gap junctions, connexin 43

## Abstract

Amniotic fluid-derived stem cells (AFSC) have been reported to differentiate into cardiomyocyte-like cells and form gap junctions when directly mixed and cultured with neonatal rat ventricular myocytes (NRVM). This study investigated whether or not culture of AFSC on the opposite side of a Transwell membrane from NRVM, allowing for contact and communication without confounding factors such as cell fusion, could direct cardiac differentiation and enhance gap junction formation. Results were compared to shared media (Transwell), conditioned media and monoculture media controls. After a 2-week culture period, AFSC did not express cardiac myosin heavy chain or troponin T in any co-culture group. Protein expression of cardiac calsequestrin 2 was up-regulated in direct transmembrane co-cultures and media control cultures compared to the other experimental groups, but all groups were up-regulated compared with undifferentiated AFSC cultures. Gap junction communication, assessed with a scrape-loading dye transfer assay, was significantly increased in direct transmembrane co-cultures compared to all other conditions. Gap junction communication corresponded with increased connexin 43 gene expression and decreased phosphorylation of connexin 43. Our results suggest that direct transmembrane co-culture does not induce cardiomyocyte differentiation of AFSC, though calsequestrin expression is increased. However, direct transmembrane co-culture does enhance connexin-43-mediated gap junction communication between AFSC.

## Introduction

Amniotic fluid has been reported to contain a population of broadly multipotent stem cells that express markers characteristic of both embryonic stem cells (ESC) and mesenchymal stem cells (MSC), can differentiate across all three germ layers, maintain prolonged undifferentiated proliferation at rates similar to ESC and do not form tumours when implanted 1–3. The existence of such cells would provide a promising new cell source for tissue engineering applications, as they have many of the advantages of both ESC and MSC, without some of the negatives, like limited proliferation of MSC or tumour formation of ESC. In addition, stem cells in amniotic fluid could provide an autologous cell source of tissue engineering repair approaches for congenital defects identified during gestation when fluid could be removed and cells collected.

Cardiac potential of AFSC has been reported upon direct co-culture with neonatal rat ventricular myocytes (NRVM), resulting in expression of cardiac markers such as sarcomere proteins, though the effect decreases when cells are separated in a Transwell system or grown in NRVM-conditioned media 1, 4, 5. Research in other progenitor cell types has found that apparent cardiac differentiation in co-culture conditions is the result of cell fusion as opposed to cell differentiation 6, 7, and this possibility has not been eliminated in AFSC studies.

Amniotic fluid-derived stem cells in mixed co-cultures with NRVM have been observed to aggregate connexin 43 at the cell membrane, suggesting the formation of functional gap junctions 4, 5. Gap junctions, transmembrane protein channels formed by connexins, with connexin 43 being the most commonly expressed in the heart 8, are responsible for the propagation of electrical signals, initiating contraction of cardiomyocytes 9. Even in the absence of functional contraction of cells in a tissue engineered cardiac construct, electrical propagation of signals through the constructs would be of the utmost importance. In cases of surgical repair of congenital heart defects, for which it may be possible to use these cells autologously, unusual electrical properties often arise in the heart around implanted patches. Patients with acellular patch repair of congenital heart defects have a 25–100 times higher chance than the general population of dying of arrhythmias 10.

**Fig. 1:**
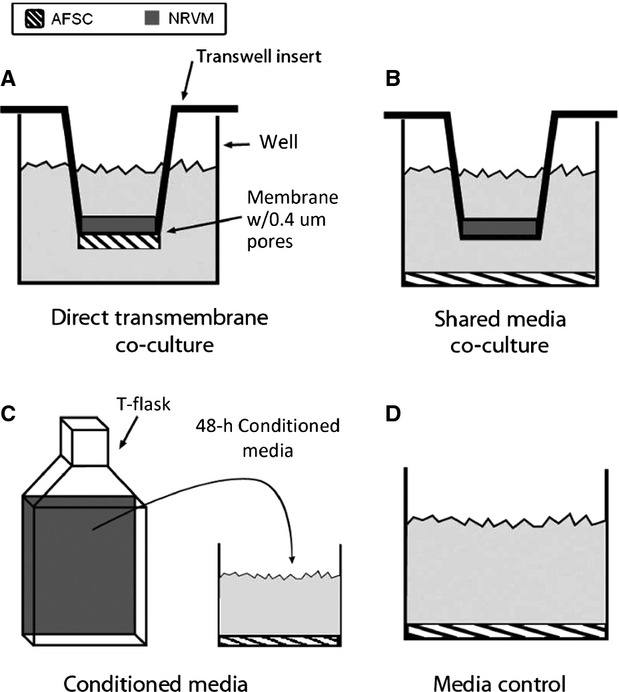
Experimental culture conditions. (**A**) In the direct transmembrane co-culture, AFSC were plated on the underside of a Transwell membrane with 0.4 μm pores and NRVM plated on the top surface of the membrane. This culture condition allowed for cell contact, but not cell fusion. (**B**) In the shared media co-culture, AFSC are plated on the bottom of wells with NRVM plated inside a Transwell insert. This culture condition allowed for secretion of factors and crosstalk by both cell types, but no cell contact. (**C**) In conditioned media cultures, NRVM were grown in T-flasks. Every 48 hrs, the media from the NRVM cultures were transferred to the AFSC cultures. This culture condition allowed AFSC to be exposed to factors secreted by the NRVM but no contact or crosstalk. (**D**) A control was performed of AFSC cultured in low-serum maintenance media.

To address the ability of AFSC to differentiate in the absence of fusion with NRVM, we cultured AFSC in NRVM-conditioned media, shared media co-cultures and in direct contact through a Transwell membrane with insufficient pore size to allow for cell fusion (Fig. 1). Through studying these culture conditions we tested whether cardiac differentiation can be induced in AFSC by NRVM in the absence of cell fusion. Furthermore, because conductive cells used in a cardiac therapy could reduce the occurrence of arrhythmias, we tested whether intercellular gap junction communication was enhanced in AFSC when co-cultured with NRVM or cultured in NRVM-conditioned media.

## Materials and methods

### AFSC isolation and cell culture

Primary human amniotic fluid was obtained from patients in their second trimester undergoing planned amnioreduction as part of a therapeutic treatment for twin-twin transfusion syndrome. Informed consent was obtained from all subjects, and the research was carried out according to the World Medical Association Declaration of Helsinki. The experimental protocol and informed consent form were approved by the Institutional Review Boards of Baylor College of Medicine and Rice University.

Undifferentiated AFSC were isolated, cultured and analysed as previously published 11. Briefly, amniotic fluid was centrifuged and cells were resuspended in a modified α-Minimum Essential Media: 63% αMEM (HyClone, Logan, UT, USA), 18% Chang Basal Medium (Irvine Scientific, Santa Ana, CA, USA), 2% Chang C supplement (Irvine Scientific), 15% foetal bovine serum (FBS; PAA Laboratories, Dartmouth, MA, USA), 1% GlutaMAX (Invitrogen, Carlsbad, CA, USA) and penicillin and streptomycin. Cells were plated at 2500 cells/cm^2^ on standard plastic Petri dishes and cultured at 37°C and 5% CO_2_ in a humidified environment. After one passage, a subpopulation of progenitor cells was isolated through fluorescence-activated cell sorting with an antibody to the membrane receptor CD117/c-Kit (BD Biosciences, Bedford, MA, USA) using a Dako MoFlo cell sorter. We have previously shown that these cells have a normal diploid male karyotype and are strongly positive for embryonic stem cell marker SSEA4; mesenchymal stem cell markers CD29, CD44, CD73, CD90 and CD105; and the immunological marker HLA-ABC, with a subpopulation (25.5%) of AFSC retaining c-kit expression through multiple passages 11. These results are comparable to those of other groups 1, 2, 4, 5, 12.

### NRVM isolation

All animal studies were performed in accordance with Rice University and Baylor College of Medicine guidelines, and experimental protocols were approved by the IACUC of each institution. Cardiomyocytes were harvested from freshly dissected ventricles of 1–3 day-old Sprague-Dawley rats using an isolation kit (Cellutron, Highland Park, NJ, USA). Cells were plated onto gelatin-coated dishes in a high-serum plating media of Dulbecco's Modified Eagle Media (DMEM; HyClone), 17% M199 (HyClone), 10% donor horse serum (PAA Laboratories), 5% FBS and penicillin and streptomycin. Approximately 24 hrs later, cells were transferred to a low-serum maintenance media of DMEM, 18.5% M199, 5% horse serum, 1% FBS, penicillin and streptomycin. Cell cultures were maintained at 37°C and 5% CO_2_ in a humidified environment. Media were changed every 2 days.

### Experimental culture conditions and set up

Amniotic fluid-derived stem cells were cultured in a direct contact, shared media and conditioned media co-culture with NRVM, as well as non-conditioned media and undifferentiated controls (Fig. 1), similar to previous co-culture studies 13. For direct transmembrane co-cultures, 5000 AFSC were plated on the underside of gelatin-coated 12-well Transwell membrane inserts (Corning, Lowell, MA, USA) and allowed to attach for 4 hrs. For shared media co-cultures, 5000 AFSC were plated in the lower well portion of the gelatin-coated Transwell system. Approximately 24 hrs later, 50,000 NRVM were plated on the top side of all inserts of Transwell cell culture dishes. For conditioned media cultures, AFSC were plated at 5000 cells per well in gelatin-coated 12-well plates and allowed to attached for ∼24 hrs before changing to NRVM-conditioned media. NRVM were plated at 80,000 cells/cm^2^ and cultured in low serum maintenance media. Conditioned media were collected from the NRVM cultures every 2 days and then used on the AFSC-conditioned media cultures. As a control, AFSC were cultured in low serum maintenance media, as described above. All groups were cultured for 14 days before analysis.

### Scrape-loading dye transfer assay

Formation of functional gap junctions between AFSC was assessed by the scrape-loading dye transfer assay as described previously 14, with some modifications. Cells were washed twice with PBS without calcium or magnesium. Cell monolayers were covered with PBS without calcium or magnesium containing 0.1% Lucifer yellow (Invitrogen), a molecule that is small enough to travel through gap junctions, but is too large to penetrate intact cell membranes, was then scrape-loaded with a scalpel. After 5 min. in the dye solution, cultures were rinsed twice with PBS containing calcium and magnesium. Cells were fixed with 4% paraformaldehyde at room temperature for 10 min. Dye transfer through gap junctions was observed and imaged along the length of the scrape using a DMI 6000B fluorescence microscope (Lieca Microsystems, Bannockburn, IL, USA). Images were assessed in a blinded fashion and measurements of lateral spreading of dye and count of cells away from scrape that dye spread were made using Image J (NIH, Bethesda, MD, USA). For each group, 12 wells were analysed, with an average of 15 images per well and 111 measurements per group.

### Immunostaining

To observe the presence and localization of proteins in the differentiated AFSC populations, differentiated cells were washed in cold (4°C) PBS and fixed with 4% paraformaldehyde (Alfa Aesar, Ward Hill, MA, USA) for 20 min. at 4°C. Cells were washed with PBS and permeabilized with 0.5% Triton X100 (CalBioChem, San Diego, CA, USA). Cells were again washed with PBS, blocked with 1% bovine serum albumin (BSA; EMD Chemicals, Gibbstown, NJ, USA) for 1 hr at 25°C and then stained overnight at 4°C with primary antibodies against the following: connexin 43/GJA1 (rabbit polyclonal; Abcam, Cambridge, MA, USA), SSEA-4 (mouse monoclonal; Santa Cruz Biotechnology, Santa Cruz, CA, USA), sarcomeric α-actinin (mouse monoclonal; Sigma-Aldrich, St. Louis, MO, USA), cardiac myosin heavy chain (mouse monoclonal; GeneTex, Irvine, CA, USA) and cardiac troponin T (rabbit polyclonal, Abcam). Cells were again rinsed with PBS then stained with DyLight-conjugated goat secondary antibodies (Jackson ImmunoResearch Laboratories, West Grove, PA, USA) diluted to 1:1000 for 30 min. at 25°C. Cells were counterstained with DAPI with VectaShield (Vector, Burlingame, CA, USA). Images were obtained using a DMI 6000B fluorescence microscope (Lieca Microsystems, Bannockburn, IL, USA).

### Quantitative reverse transcription – polymerase chain reaction

All reagents for qRT-PCR were purchased from Applied Biosystems (Carlsbad, CA, USA), except the RNeasy Mini Kit from Qaigen. Total RNA was extracted from cultured cells using the RNeasy Mini Kit, according to the manufacturer's protocol. DNA was removed from all samples using the DNA-free kit, according to manufacturer's protocol. cDNA was prepared from 450 ng total RNA, reverse transcribed in a 30 μl reaction mix using the High Capacity cDNA Reverse Transcription Kit, according to manufacturer's protocol. Relative quantification of connexin 37/GJA4, connexin 40/GJA5, connexin 43/GJA1, connexin 45/GJC1 and GATA-4 were performed using TaqMan Gene Expression Assays Hs00704917_s1, Hs00270925_s1, Hs00748445_s1, Hs00271416_s1 and Hs00171403_m1 respectively. Expression was normalized to GAPDH using TaqMan Gene Expression Assay Hs99999905_m1. Briefly, 15 ng cDNA was amplified in TaqMan Gene Expression Master Mix with 250 nM TaqMan probe in a 20 μl reaction using the Standard program for 60 cycles on an ABI ViiA 7 Real-Time PCR System. RNA was extracted from nine total culture wells, over three separate experimental trials and all samples were run in four replicates. Human cardiac RNA was isolated from a sample removed as part of a surgical treatment and was collected under an IRB approved protocol from Baylor College of Medicine and Rice University and used as a positive control. Data were analysed using the comparative C_T_ method with software from Applied Biosystems, with all samples normalized to GAPDH and an undifferentiated AFSC sample in the case of connexins and the human cardiac tissue for the GATA-4 analysis.

### Western blot

Western blot electrophoresis and transfer materials were purchased from Bio-Rad (Hercules, CA, USA), and developing materials were purchased from Thermo Scientific (Rockford, IL, USA). After 14 days of differentiation, total protein lysates were isolated from each experimental culture group in RIPA buffer with protease and phosphotase inhibitor cocktails (Thermo Scientific). Lysate concentrations were analysed using a bicinchoninic acid kit (BCA; Thermo Scientific). Extracts were denatured using β-mercaptoethanol and boiling for 5 min., then diluted to equal concentrations of total protein. The samples were electrophoresed by 0.1% sodium dodecyl sulphate-polyacrylamide gel electrophoresis (SDS-PAGE) and transferred onto nitrocellulose membranes at 100 V for 1.5 and 1.0 hrs respectively. Membranes were washed in Tris-buffered saline with 0.05% Tween-20 (TBST), then blocked with 5% non-fat milk in TBST to reduced non-specific binding. Membranes were incubated overnight at 4°C with primary antibodies: Connexin 43 (Abcam), Cx43 phosphorylated at residue serine 368 (pCx43s368; Cell Signaling Technology, Beverly, MA, USA), calsequestrin (Abcam) and GAPDH (Abcam). Membranes were washed and incubated for 30 min. at 25°C with secondary antibodies conjugated to horseradish peroxidase (Jackson ImmunoResearch Laboratories). A 1-minute Luminol reagent exposure was used to provide chemiluminescence, and images were developed using high-sensitivity x-ray film. Western blots were normalized to GAPDH expression. Western blot analysis was performed using Image J (NIH).

### Intracellular flow cytometry

After 14 days of differentiation, cells were detached from culture dishes using 0.125% trypsin (HyClone). Cells were then resuspended in 4% paraformaldehyde (Alfa Aesar) and refrigerated for 1 hr. Cells were then centrifuged, supernatant removed, resuspended in PBS and stored at 4°C for future staining. For staining for intracellular flow cytometry, cells were permeabilized with 0.5% Tween 20 in PBS at 37°C for 15 min. Cells were rinsed with PBS, then blocked with 10% FBS in PBS containing 0.5% Tween 20 at room temperature for 5 min. Cells were then incubated at room temperature for 30 min. with the primary antibody: calsequestrin-2 (Santa Cruz Biotechnology). Samples were washed twice with PBS containing 10% FBS and 0.5% Tween 20 then incubated with PE-conjugated goat anti-rabbit secondary antibody (Jackson ImmunoResearch Laboratories) for 30 min. at room temperature. After samples were again washed twice they were analysed using a BD LSRII flow cytometer. FACSDiva software (BD Biosciences) was used for all flow cytometry data collection. FlowJo software (Tree Star, Inc., Ashland, OR, USA) was used for data analysis.

### Statistical analysis

Results are presented as means ± SD. Sample numbers for each experiment are represented in their respective figures. One-way anova and Student's *t*-tests with a Bonferroni correction for multiple comparisons were used to test for significant differences. Significance was determined as *P* < 0.05.

## Results

### Markers of cardiac differentiation

**Fig. 2:**
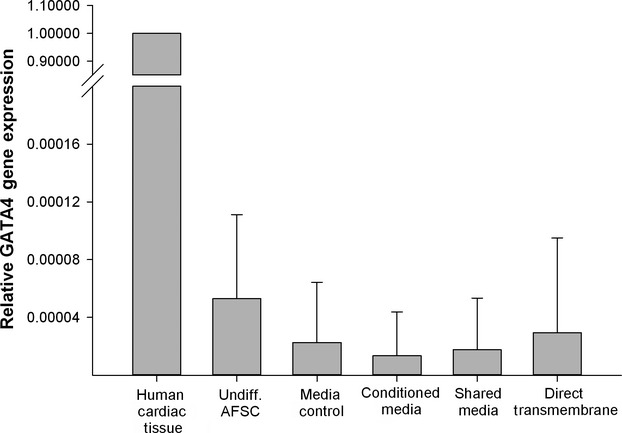
GATA-4 gene expression. Gene expression analysis *via* qRT-PCR showed that all experimental conditions have GATA-4 gene expression levels that are not significantly different from zero. All samples were compared relative to RNA extracted from a human right ventricular outflow tract sample (*n* = 2 for undifferentiated AFSC, 4 for media control, and 5 for all other experimental samples).

Amniotic fluid-derived stem cells did not express the cardiac transcription factor GATA-4 in any culture conditions (Fig. 2) and little to no GATA-4 expression was observed in Western blots (Supporting Information Figure S1). No co-culture conditions showed positive staining for the late-stage cardiac sarcomere proteins cardiac myosin heavy chain, cardiac troponin T and sarcomeric α-actinin (results not pictured).

**Fig. 3:**
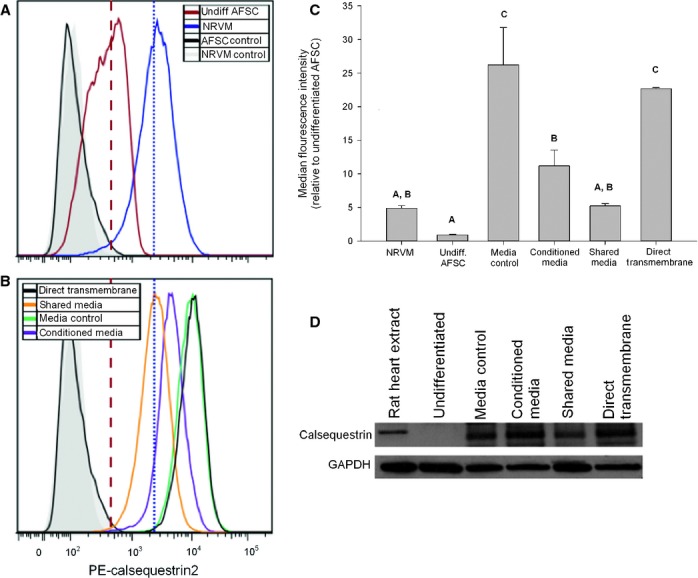
Calsequestrin 2 protein expression. Representative histograms of flow cytometry results for undifferentiated AFSC and cultured NRVM (**A**) and experimental samples (**B**). The dashed lines indicate the median fluorescence intensities of undifferentiated AFSC (red) and NRVM (blue). Relative median fluorescence intensity, representing relative protein expression, was significantly higher in the media control and direct transmembrane cultures compared to all other groups (*P* ≤ 0.002; **C**). All bars that do not share at least one letter label in common have significant statistical difference. Protein expression of calsequestrin was confirmed *via* Western blots (**D**). For flow cytometry experiments, *n* = 2 for direct transmembrane co-culture and 4 for all other groups; 100,000 cells were analysed per sample.

Calsequestrin 2, the cardiac-specific isoform of the protein that stores calcium ions in the sarcoplasmic reticulum, was analysed through flow cytometry. All samples had calsequestrin 2 expression above background; therefore, comparisons were done using the median fluorescence intensity of each sample, normalized to the undifferentiated AFSC median fluorescence intensity. Calsequestrin 2 was increased in the maintenance media control and direct transmembrane co-cultures compared to the relative median fluorescence intensities of all other samples (26.238 ± 5.542 and 22.677 ± 0.156 for media control and direct transmembrane, respectively, *versus* 4.908 ± 0.389, 0.931 ± 0.099, 11.199 ± 2.352 and 5.242 ± 0.318 for NRVM, undifferentiated AFSC, conditioned media and shared media co-cultures respectively; *P* ≤ 0.002; Fig. 3). Conditioned media cultures also showed an elevated level of calsequestrin 2 compared to undifferentiated AFSC (*P* < 0.001).

**Fig. 4:**
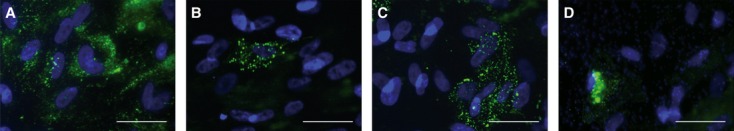
SSEA4 protein expression. (**A**) SSEA4 expression was maintained in AFSC after culture in the maintenance media control for 2 weeks. Expression was reduced in (**B**) conditioned media cultures, (**C**) shared media co-cultures, and (**D**) direct transmembrane co-cultures. Scale bars: 50 μm.

Undifferentiated AFSC showed expression of SSEA4, an embryonic stem cell marker, in both flow cytometry and immunostaining. After 2 weeks in culture, the maintenance media control group maintained SSEA4 expression in immunostained samples. By contrast, the conditioned media, shared media co-culture and direct transmembrane co-culture groups showed a decreased level of SSEA4, observed through immunostaining (Fig. 4).

### Intercellular gap junction communication

**Fig. 5:**
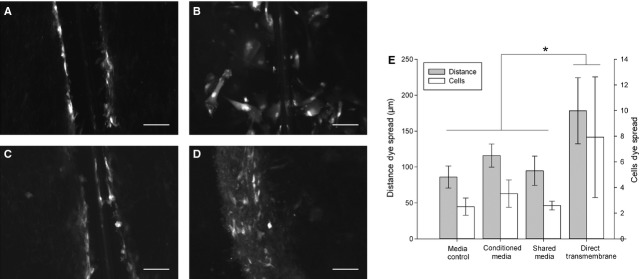
Diffusion of Lucifer yellow dye between cells through gap junctions. A scrape-loading dye transfer assay was performed on confluent cultures, which allowed for Lucifer yellow to enter damaged cells along the scrape, and then diffuse between cells through gap junctions. Wells were imaged along the length of scrapes and the distance of dye diffusion from the scrape was measured as a representation of gap junction functionality. Representative images of (**A**) maintenance media control, (**B**) conditioned media, (**C**) shared media co-culture and (**D**) direct transmembrane co-culture. Measurements of distance of dye diffusion and number of cells dye diffused through found all groups to be significantly different from direct transmembrane co-cultures (*P < 0.001). *N* = 12 for all groups. Scale bars: 100 μm.

Amniotic fluid-derived stem cells cultured in a direct transmembrane co-culture with NRVM had significantly higher dye diffusion distance than all other groups (178 ± 46 *versus* 95 ± 20, 116 ± 16 and 86 ± 15 μm for direct transmembrane, shared media, conditioned media and maintenance media control respectively; *P* < 0.001; Fig. 5). In addition, the dye diffused through an increased number of cells in the direct transmembrane co-culture compared with the other experimental groups (7.92 ± 4.71 *versus* 2.59 ± 0.34, 3.52 ± 1.07 and 2.50 ± 0.67 cells for direct transmembrane, shared media, conditioned media and maintenance media control respectively; *P* < 0.001). These increased measures of dye diffusion indicate an increased level of intercellular gap junction formation and communication in the direct transmembrane co-cultures.

### Expression and localization of connexin

To elucidate possible mechanisms for increased functional gap junction formation, expression levels of connexin genes commonly found in cardiac tissue were analysed.

**Fig. 6:**
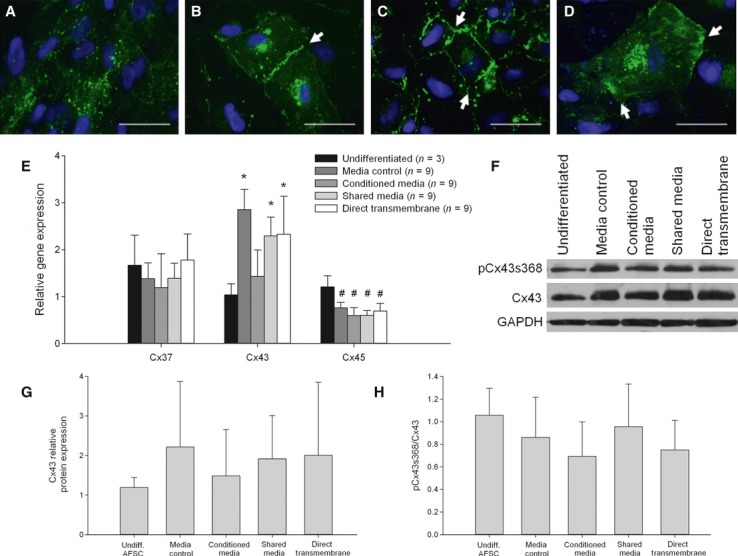
Connexin gene and protein expression and cellular location. (**A**) Cx43 immunostaining of maintenance media control shows no localization of Cx43 to the cell membranes. Localization of Cx43 to cell membranes and junctions between cells is observed in (**B**) conditioned media cultures, (**C**) shared media co-cultures and (**D**) direct transmembrane co-cultures. Gene expression analysis (**E**) *via* qRT-PCR showed no difference between groups for Cx37. Cx43 gene expression was significantly increased in media control, shared media co-culture and direct transmembrane co-culture compared to undifferentiated AFSC (*P < 0.05). Cx45 gene expression was significantly decreased in all groups compared to undifferentiated AFSC (#*P* < 0.001). Western blots were performed for Cx43 and pCx43s368, and representative blots are shown (**F**). Relative Cx43 protein expression was not significantly different between any groups, but was correlated with gene expression (G; *n* = 3 for undifferentiated AFSC; *n* = 7 for all experimental groups). pCx43s368/Cx43 ratio was not significantly different between groups, but was inversely associated with dye diffusion (H; *n* = 3 for undifferentiated AFSC; *n* = 7 for all experimental groups). Scale bars: 50 μm.

Immunostaining for connexin 43 showed localization to the membrane in the conditioned media, shared media co-culture and direct transmembrane co-culture groups. No localization of connexin 43 to the cell membrane was observed in the maintenance media control or undifferentiated AFSC (Fig. 6A–D).

Connexin 43 gene expression was significantly increased in direct transmembrane co-culture compared with undifferentiated AFSC (2.33 ± 0.81 *versus* 1.04 ± 0.24 normalized to undifferentiated AFSC control, *P* = 0.016; Fig. 6E). Connexin 43 expression was also increased in maintenance media control (2.86 ± 0.43, *P* < 0.001), shared media co-culture (2.29 ± 0.40, *P* = 0.021), but not in conditioned media cultures (1.43 ± 0.57). Connexin 45 expression in all experimental cultures was significantly lower than that in the undifferentiated AFSC (*P* < 0.001, Fig. 6E). Connexin 37 was not found to be significantly different across undifferentiated AFSC or any of the experiment culture groups, by a one-way anova (*P* = 0.2, Fig. 6E). Connexin 40 expression in all cultures was too low to be consistently amplified under the qRT-PCR conditions used and results were not analysed.

Protein levels of connexin 43, analysed by Western blotting, were correlated with gene expression, but were not statistically different across samples (anova, *P* = 0.79, Fig. 6G). The fraction of pCx43s368/Cx43 was inversely correlated with dye transfer lengths, but not statistically different between samples (anova, *P* = 0.39, Fig. 6H).

## Discussion

Our experiments have shown that cell contact between NRVM and AFSC produces greater functional gap junction formation than co-culture systems without cell contact. Increased gap junction formation likely results from increased connexin 43 expression, decreased phosphorylation of connexin 43, and increased homing of connexin 43 to the cell membrane. Despite the increased in gap junctions in this culture system, co-cultures of AFSC with NRVM in a manner that does not allow for cell fusion were insufficient for inducing complete cardiac differentiation of AFSC, as demonstrated by the lack of cardiac transcription factor GATA-4 and cardiac sarcomere proteins.

The lack of cardiac sarcomere protein expression in AFSC co-cultured with NRVM in this study is consistent with other conditioned or shared media studies and suggests that full differentiation either requires full cell-cell contact or cell fusion. Previous studies have found that direct mixing of AFSC and NRVM results in a small population of cells expression both human protein markers or cell labels and late-stage cardiac markers troponin T, troponin I, or α-actinin 1, 4, 5. In direct transmembrane co-culture conditions, which allowed for cell contact but did not allow for cell fusion or exchange of nuclei between cells, we were unable to observe expression of myosin heavy chain or troponin T after 14 days in culture. This result is consistent with conditioned media results in previous studies, which also demonstrated no expression of myosin heavy chain or troponin T that could be verified by gene expression or protein content 1.

Although calsequestrin was observed to be up-regulated in some experimental groups, this increase was not present in all groups. The upregulation of calsequestrin in the absence of additional markers of cardiac differentiation re-emphasizes that the expression of several cardiac proteins is necessary to confirm a cardiac phenotype in differentiated cells. The lack of late-stage cardiac sarcomere proteins suggests that close proximity cell culture and limited cell contact is insufficient to induce complete cardiac differentiation.

The membrane aggregation of connexin proteins in direct transmembrane co-cultures leads to functional gap junctions. Scrape-loading dye transfer assay demonstrated that AFSC in direct contact co-cultures had significantly stronger gap junction communication than in other types of culture, and immunostaining of connexin 43 verified that AFSC in direct contact co-culture had significant connexin aggregation at the cell membrane. This result mirrors immunohistochemical results in co-cultured AFSC in previous studies 4, 5. In addition, though trending and not statistically significant, conditioned media AFSC cultures had an increase in gap junction communication compared to maintenance media control and this increase was not apparent in the shared media co-cultures. This result suggests that cross-talk between AFSC and NRVM in culture may have a significant effect on the behaviour and possibly cytokine release of each cell type.

Changes in gene expression of common cardiovascular gap junction proteins suggest the involvement of connexin 43, but cannot fully explain gap junction functionality. The dominant connexins expressed in cardiovascular tissue are connexin 37, 40, 43 and 45 15. Of these, the only gene to show significant upregulation in differentiated AFSC compared to undifferentiated controls was connexin 43, which increased in every tested condition except conditioned media. The lack of correlation between gene expression levels and amount of intercellular gap junction communication indicates that gene expression alone cannot explain the variations in the formation of gap junctions. Analysis of post-translational modification or trafficking of gap junction proteins may be necessary to elucidate the mechanism underlying the functionality variations. Interestingly, the ratio of phosphorylated connexin 43 at serine 368, which has been shown to mark the protein for intercellular location or degradation 16, 17, is inversely related with observed gap junction functionality and could suggest a possible mechanism for lower gap junction activity, independent of connexin 43 gene expression, in shared media co-cultures.

Statistical analysis of PCR samples from different experimental trials revealed that every co-culture condition had significant variation in the expression levels of at least one connexin gene between experiments using different batches of NRVM. This significant difference suggests that one source of gene expression level variation is the isolation of primary NRVM, which is another reason that differentiation methods dependent on NRVM may be less ideal. Other differentiation methods that are not cell dependent would likely allow for increased reproducibility of results and additional factors could be added to induce production of cardiac sarcomere proteins.

The ease of isolation, rapid proliferation rate and broadly multipotent nature of AFSC make them a promising cell source for tissue engineering. This study demonstrates the capability of AFSC to create functional gap junctions. Although no cardiac sarcomere proteins were observed in any of the culture conditions tested, AFSC demonstrated potential in cardiac applications because of their ability to form functional gap junctions. However, additional cues, or fusion of AFSC with cardiomyocytes, are likely required for complete cardiac differentiation.

## Supporting information

**Figure S1** Representative Western blots of GATA4 protein expression in rat heart lysate, undifferentiated AFSC, maintenance media control, conditioned media, shared media co-cultures and direct transmembrane co-cultures.

## References

[1] Chiavegato A, Bollini S, Pozzobon M (2007). Human amniotic fluid-derived stem cells are rejected after transplantation in the myocardium of normal, ischemic, immuno-suppressed or immuno-deficient rat. J Mol Cell Cardiol.

[2] De Coppi P, Bartsch G, Siddiqui MM (2007). Isolation of amniotic stem cell lines with potential for therapy. Nat Biotechnol.

[3] Tsai MS, Lee JL, Chang YJ (2004). Isolation of human multipotent mesenchymal stem cells from second-trimester amniotic fluid using a novel two-stage culture protocol. Hum Reprod.

[4] Guan X, Delo DM, Atala A (2010). *In vitro* cardiomyogenic potential of human amniotic fluid stem cells. J Tissue Eng Regen Med.

[5] Yeh YC, Wei HJ, Lee WY (2010). Cellular cardiomyoplasty with human amniotic fluid stem cells: *in vitro* and *in vivo* studies. Tissue Eng Part A.

[6] Alvarez-Dolado M, Pardal R, Garcia-Verdugo JM (2003). Fusion of bone-marrow-derived cells with Purkinje neurons, cardiomyocytes and hepatocytes. Nature.

[7] Nygren JM, Jovinge S, Breitbach M (2004). Bone marrow-derived hematopoietic cells generate cardiomyocytes at a low frequency through cell fusion, but not transdifferentiation. Nat Med.

[8] Marquez-Rosado L, Solan JL, Dunn CA (2012). Connexin43 phosphorylation in brain, cardiac, endothelial and epithelial tissues. Biochim Biophys Acta.

[9] Rohr S (2004). Role of gap junctions in the propagation of the cardiac action potential. Cardiovasc Res.

[10] Silka MJ, Hardy BG, Menashe VD (1998). A population-based prospective evaluation of risk of sudden cardiac death after operation for common congenital heart defects. J Am Coll Cardiol.

[11] Benavides OM, Petsche JJ, Moise KJ (2012). VEGF induces endothelial differentiation of amniotic fluid-derived stem cells. Tissue Eng Part A.

[12] Zhang P, Baxter J, Vinod K (2009). Endothelial differentiation of amniotic fluid-derived stem cells: synergism of biochemical and shear force stimuli. Stem Cells Dev.

[13] Jacot JG, Wong JY (2008). Endothelial injury induces vascular smooth muscle cell proliferation in highly localized regions of a direct contact co-culture system. Cell Biochem Biophys.

[14] el-Fouly MH, Trosko JE, Chang CC (1987). Scrape-loading and dye transfer. A rapid and simple technique to study gap junctional intercellular communication. Exp Cell Res.

[15] Duffy HS, Fort AG, Spray DC (2006). Cardiac connexins: genes to nexus. Adv Cardiol.

[16] Boublik J, Park H, Radisic M (2005). Mechanical properties and remodeling of hybrid cardiac constructs made from heart cells, fibrin, and biodegradable, elastomeric knitted fabric. Tissue Eng.

[17] Black LD, Meyers JD, Weinbaum JS (2009). Cell-induced alignment augments twitch force in fibrin gel-based engineered myocardium *via* gap junction modification. Tissue Eng Part A.

