# Supplementary figures and images for: Formation of functional gap junctions in amniotic fluid-derived stem cells induced by transmembrane co-culture with neonatal rat cardiomyocytes
Source: J Cell Mol Med. 2013 May 2;17(6):774–81. doi: 10.1111/jcmm.12056 (PMC3823181; doi:10.1111/jcmm.12056)

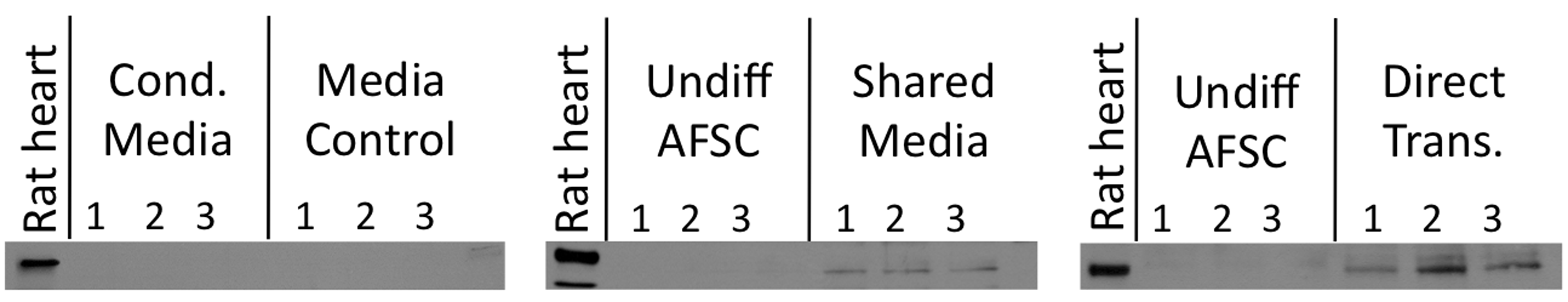

Supplement: Supplementary file 1 [file jcmm0017-0774-SD1.tiff]
